# The Added Value of Whole-Exome Sequencing for Anomalous Fetuses With Detailed Prenatal Ultrasound and Postnatal Phenotype

**DOI:** 10.3389/fgene.2021.627204

**Published:** 2021-07-22

**Authors:** Miao He, Liu Du, Hongning Xie, Lihe Zhang, Yujun Gu, Ting Lei, Ju Zheng, Dan Chen

**Affiliations:** ^1^Department of Ultrasonic Medicine, Fetal Medical Center, The First Affiliated Hospital of Sun Yat-sen University, Guangzhou, China; ^2^Guangzhou Kingmed Diagnostics Group, Guangzhou, China

**Keywords:** whole-exome sequencing, anomalous fetuses, prenatal ultrasound, postnatal phenotype, genetic variants

## Abstract

**Objectives:**

The objective of the study was to explore the added value of whole-exome sequencing (WES) in abnormal fetuses with detailed prenatal ultrasound and postnatal phenotype with normal karyotype and chromosomal microarray analysis (CMA).

**Methods:**

Parents of fetuses with structural abnormalities by prenatal ultrasound who consented to provide fetal samples were prospectively recruited from January 2017 to December 2019. With aneuploidies or cases with copy number variations (CNVs) excluded, WES was performed for cases with normal karyotype and CMA results. Detailed prenatal ultrasound and postnatal imaging or pathology features were recommended for further interpretation of genetic variants.

**Results:**

WES was performed for 94 eligible fetuses, DNA samples of which were extracted from 53 parent–fetus trios and 41 proband-only fetal tissues. A diagnostic genetic variant was identified in 37 (39.4%) of 94 fetuses, and 34 (64.2%) were detected in 53 trios, which was significantly greater than 3 (7.3%) in 41 proband-only cases (*p* < 0.001). In 34 trios with diagnostic genetic variants, 23 (67.6%) were *de novo* and 11 (32.4%) were inherited with two homozygous and nine heterozygous variants. Fourteen (14.9%) of 94 fetuses had a variant of uncertain significance (VUS). Among 94 cases, six affected pregnancies continued and 88 terminated, and 57 of 88 terminated cases underwent postmortem examinations. With accurate phenotypes demonstrated by prenatal ultrasound and postnatal autopsies, the clinical phenotypes were correlated in 33 (89.2%) of 37 cases with specific genotypes, with the highest matching ratio in skeletal diseases (20/33, 60.6%).

**Conclusion:**

WES has added value in the genetic diagnosis of abnormal fetuses with normal karyotypes and CMA, particularly in skeletal diseases. Using WES in various anomalous fetuses can broaden the understanding of prenatal phenotypes and genetic variants.

## Introduction

Congenital anomalies affect 2–4% of all infants and are responsible for 21% of perinatal deaths (Ely and Driscoll, 2019). Using ultrasound, these abnormalities can be identified in the first and second trimesters, ranging from fetal hydrops to major lethal disorders. Although the standard genetic investigation of fetal abnormalities, karyotype, and/or chromosomal microarray analysis (CMA) is applied for aneuploidies and copy number variations (CNVs), greater than 60% of pregnancies do not have a genomic diagnosis to guide future care and genetic counseling (Petrovski et al., 2019).

Prenatal whole-exome sequencing (WES) is a useful and valuable tool for the genetic diagnosis of fetal anomalies with a diagnostic yield between 6.2 and 81% (Carss et al., 2014; Alamillo et al., 2015; Drury et al., 2015; Yates et al., 2017; Chandler et al., 2018; Yadava and Ashkinadze, 2019). The underlying etiology has been identified in many fetal abnormalities by the use of WES, including anomalies of the kidney and urinary tract (13%) (Lei et al., 2017), skeletal disease (88.9%) (Han et al., 2020), and non-immune hydrops fetalis (29%) (Sparks et al., 2020); however, the limited prenatal phenotype information, difficulties in variant interpretation of secondary findings, and the time-consuming nature of this costly technology have put off the widespread clinical implementation of WES. To expand the prenatal phenotype–genotype spectrum and facilitate the utility of prenatal WES, we analyze the diagnostic yield of WES in fetuses with different kinds of abnormalities with detailed ultrasound and postnatal phenotypes.

## Materials and Methods

### Participants and Sample Collection

From January 2017 to December 2019, parents of fetuses with structural anomalies detected by prenatal ultrasound were invited to participate in this study at The First Affiliated Hospital of Sun Yat-sen University. Prenatal ultrasound, including a detailed ultrasound scan and the assessment of fetal biometry, was performed by an expert with 30 years of experience in the obstetrics ultrasound field. In the cases of termination of pregnancy (TOP), postmortem examinations were performed as much as possible after obtaining permission of parents to access detailed and accurate clinical phenotypes. When autopsy findings or phenotypes differed from those of prenatal ultrasound, clinical manifestations were corrected to optimize the interpretation of genetic variants. In this study, singleton pregnancies were required, and cases with available fetal samples were included. A detailed pre-test counseling was offered for these parents. Cases with anomalies in the first trimester and fetuses with aneuploidies or CNVs were excluded. Fetuses with a known family history of genetic mutation or a known infection or exposure to a known teratogenic drug were excluded.

After prenatal ultrasound diagnosis and pre-test counseling, if parents wanted to continue the pregnancy, amniocentesis or cord blood sampling was collected for genetic testing according to the fetal gestational age at the time of invasive procedures. Karyotype and CMA were performed first, and then WES was carried out for cases with normal karyotypes and CMA by using the same sample. In the cases of fetal demise or pregnancy termination, fetal tissue samples were collected for testing after termination. Parents were encouraged to provide blood samples for subsequent testing to assist in the interpretation of genetic variants. Samples of the abnormal fetus, mother, and father all collected in one case, were termed the parent–fetus trios. Informed consent was obtained from all parents, and they were informed that only results related to clinical phenotypes detected by ultrasound and autopsy would be reported back, not secondary findings. The turnaround time was 6–8 weeks, including reports of trios. This study was approved by the Ethics Committee of The First Affiliated Hospital of Sun Yat-sen University.

### Whole-Exome Sequencing

Genomic DNA was extracted with a QIAamp DNA Blood Mini kit (Qiagen, Hilden, Germany) following the manufacturer’s protocol. The extracted DNA was fragmented randomly and then purified using the magnetic particle method. DNA fragments were ligated with adaptors and captured by probes of the IDT XGen Exome Research Panel (IDT, Lowa, United States) targeting approximately 19,396 genes. The DNA libraries, after enrichment and purification, were sequenced on a NovaSeq 6000 sequencer according to the manufacturer’s instructions (Illumina, San Diego, CA, United States). All reads were aligned to the reference human genome (UCSC hg19) by Burrows–Wheeler Aligner (BWA) (v.0.5.9-r16) (Li and Durbin, 2010). After data annotation using the PriVar toolkit (Zhang et al., 2013), the clinical significance of the variants was identified (Yang et al., 2013). Databases such as OMIM^1^, GeneCards^2^, ClinVar^3^, and Human Gene Mutation Database^4^ were used to determine pathogenicity. The detected variants were confirmed using PCR, and the products were subjected to direct sequencing on a 3500XL Genetic Analyzer (Applied Biosystems, Foster City, CA, United States) according to the manufacturer’s instructions. Each variant was categorized by the performing laboratory as pathogenic, likely pathogenic, variants of uncertain significance (VUS), likely benign, or benign according to the American College of Medical Genetics and Genomics (ACMG) guidelines (Richards et al., 2015).

### Statistical Analysis

The number of pathogenic and likely pathogenic variants in the trios and probands and the number of diagnostic variants in phenotype classes were compared with the chi-square test. A value of *p* < 0.05 was used to define statistical significance, and the tests were two-tailed. The statistical analysis was performed with SPSS (version 25.0).

## Results

### Demographic Characteristics

**FIGURE 1:**
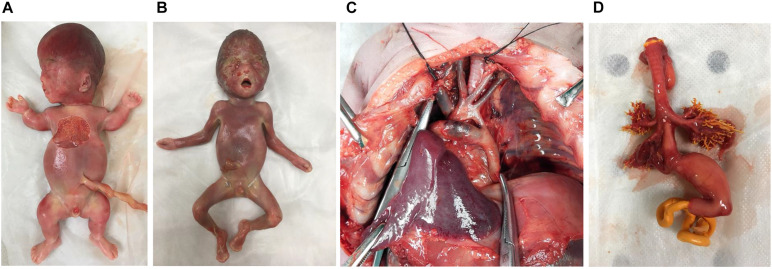
The postmortem phenotypes of four cases. **(A)** Case 16. Thanatophoric dysplasia (type I), fetus with frontal bossing, thoracic hypoplasia, and shortened bone (<-6 to 8 SD) with pathogenic missense variant in *FGFR3*. **(B)** Case 29. Apert syndrome, fetus with depressed nasal bridge, turricephaly, and brachy-syndactyly of hands and feet with pathogenic missense variant in *FGFR2*. **(C)** Case 43. Coarctation of the aorta, fetus with coarctation of the aorta with variant of uncertain significance (*CITED2*). **(D)** Case 10. Esophago-tracheal fistula, fetus with persistent left superior vena cava, esophago-tracheal fistula and polyhydramnios without diagnostic genetic variant.

From January 2017 to December 2019, 102 couples with fetuses that had structural anomalies consented to participate in our study. Eight cases with pathogenic CNVs were excluded, and 94 eligible cases with normal karyotyping and CMA results underwent WES. The median maternal age was 31 years (range 22–42 years). Gestational age (GA) at ultrasound screening ranged from 16 to 37 weeks (median GA 25 + 1 weeks). Six couples continued the pregnancies, and 88 chose to terminate. Postmortem examinations were done for 57 (57/88, 64.8%) cases. The postmortem phenotypes of four cases with different types of genetic results are shown in Figure 1. The clinical outcomes of the remaining 31 cases were known through telephone tracts without postmortem examination.

### Phenotypes

Accurate phenotypic information was obtained from prenatal ultrasound and postnatal necropsy/postnatal imaging [fetal magnetic resonance imaging (MRI) and X-ray after termination]. Of the 57 autopsy cases, the diagnostic correction was only made in case 89 with unilateral anophthalmia, and unilateral microphthalmia was confirmed after postmortem examination. Case 80 with asymmetric brain dysplasia was detailed as lissencephaly of the right cerebral hemisphere by pathological analysis. The postnatal phenotypes of the remaining cases were consistent with prenatal ultrasound phenotypes. The 94 cases were categorized into 11 classes according to the anatomical systems involved (Table 1). There were 60 cases (60/94, 63.8%) that affected one anatomic system and 34 cases (34/94, 36.2%) with anomalies that affected two or more anatomic systems. The phenotypes included various kinds of abnormalities as shown in examples of specific findings in Table 1.

**TABLE 1:** The frequency of fetal anomalies and examples of specific findings.

Category of anomaly	Numbers of fetuses (*n* = 94)	Examples of specific findings
Skeletal system	36 (38.3%)	Shortened and bowing bone, dysplasia of thoracic vertebra and ribs, hemivertebra, scoliosis, syndactyly
Cardiovascular system	26 (27.7%)	Tetralogy of Fallot, interrupted aortic arch, transposition of the great arteries, pulmonary artery sling
Central nervous system	21 (22.3%)	Agenesis of corpus callosum, Dandy–Walker malformation, pachygyria, cerebral dysplasia, hydrocephalus
Genitourinary system	19 (20.2%)	Enlarged polycystic and echogenic kidneys, hypospadias, common cloacal deformity
Neuromuscular system	14 (14.9%)	Clubfeet, arthrogryposis
Head	12 (12.8%)	Micrognathia, bilateral anophthalmia, unilateral microphthalmia, depressed nasal bridge
Hydrops	5 (5.3%)	Hydrops, pleural effusion
Gastrointestinal system	4 (4.3%)	Esophago-tracheal fistula, mesenteric cyst, situs in vs.
Growth abnormality	3 (3.2%)	Intrauterine growth restriction
Abdominal wall	2 (2.1%)	Omphalocele, exstrophy of bladder
Respiratory system	1 (1.1%)	Dysplasia of right lung

*Fetuses with several anomalies involving different anatomic systems were counted several times.*

### Genetic Variants

In our prospective cohort, DNA samples were obtained from 76 fetal tissues, 12 amniocenteses, and 6 cord blood samples. The diagnostic yield of WES was 39.4% (37/94) with likely pathogenic (19/94, 20.2%) and pathogenic variants (18/94, 19.1%). VUS was found in 14 cases (14/94, 14.9%) with potential clinical usefulness. Of the 94 fetuses who underwent WES, 53 were trios, and 41 were proband-only cases. The diagnostic yield of pathogenic variants in trios (34/53, 64.2%) was significantly higher than that in probands (3/41, 7.3%) (*p* < 0.001). In 34 trios with diagnostic genetic variants, 23 (23/34, 67.6%) were *de novo*, and 11 (11/34, 32.4%) were inherited with two homozygous and nine heterozygous variants. Maternal carriers were found in four cases, paternal carriers were found in two cases, and carriers of both parents were found in five cases. The ultrasonic phenotype and diagnostic genetic variants of 37 cases are shown in Table 2. Of the 37 cases with diagnostic genetic variants, the clinical phenotypes were correlated with genotypes in 33 cases (89.2%) with the highest matching ratio in skeletal diseases (20/33, 60.6%), such as *FGFR3* in the case with short bones considering achondroplasia, *FGFR2* in the case with turricephaly, brachy-syndactyly of hands and feet considering Apert syndrome, and *COL1A1* or *COL1A2* in the case with short and angulated bones considering osteogenesis imperfecta. The remaining four cases had incidental findings that could expand the phenotype–genotype spectrum, including cardiac anomalies with *ANKRD11*, central nervous system anomalies with *FGFR2*, multisystem anomalies with *C5orf42*, and genitourinary anomalies with *ETFDH*.

**TABLE 2:** Phenotypes and pathogenic variants of 37 cases by WES.

Case ID	Prenatal imaging phenotype	Gene	Alternation	Consequence	Inheritance	ACMG classification	Origin (inherited/*de novo*)	Novel or previously reported (PMID)	Associated clinical condition
2	AMC, micrognathia, polyhydramnios	*MYH3*	c.2015G > A, p. Arg672His	Missense	AD; heterozygous	Pathogenic (PS3 + PM2 + PP2 + PP3 + PP4 + PP5)	*De novo*	AMC (25740846)	AMC
3	VSD, IAA (type B)	*ANKRD11*	c.576_579dupAGAC, p. Val194fs	Frameshift	AD; heterozygous	Likely pathogenic (PM2 + PM4 + PP3 + PP5)	*De novo*	Novel	KBG syndrome
4	Shortened and bowing bone (−2−−3 SD)	*COL1A2*	c.2565 + 1G > A, P.?	Splicing mutation	AD; heterozygous	Pathogenic (PVS1 + PS2 + PM4 + PP3 + PP4 + PP5)	*De novo*	Osteogenesis imperfecta (16705691)	Osteogenesis imperfecta
9	Low nasal bridge, micrognathia, enlarged left ventricular system, ARSA, clenched hands	*KAT6B*	c.5385C > A, p. Tyr1795*	Non-sense	AD; heterozygous	Likely pathogenic (PS2 + PM2 + PM4)	*De novo*	Novel	SBBYSS syndrome
13	sacral vertebra defects, fusion of vertebra and ribs	*MESP2*	c.718delG, p. Gly240fs	Frameshift	AR, homozygous	Likely pathogenic (PM2 + PM4 + PP3 + PP55)	Parental inherited	Novel	Spondylocostal dysostosis
14	VSD, IAA (type B), duplex kidney	*USP9X*	c.3502C > T, p. Arg1168*	Non-sense	X-linked; heterozygous	Likely pathogenic (PVS1 + PM2 + PM4)	−	Novel	Intellectual disability
16	Frontal bossing, thoracic hypoplasia, shortened bone (< −6SD−8SD)	*FGFR3*	c.1118A > G, p. Tyr373Cys	Missense	AD; heterozygous	Pathogenic (PS2 + PM2 + PP2 + PP3 + PP4 + PP5)	*De novo*	Thanatophoric dysplasia type I (8845844)	Thanatophoric dysplasia
17	Bowing femur bone	*COL1A2*	c.587G > T, p. Gly196Val	Missense	AD; heterozygous	Pathogenic (PS2 + PM2 + PP2 + PP3 + PP4 + PP5)	*De novo*	Osteogenesis imperfecta (23692737)	Osteogenesis imperfecta
19	Pachygyria, bilateral ventriculomegaly, spina bifida occulta, multiple vertebra hypoplasia, fusion of vertebral arch	*FGFR2*	c.1052C > G, p. Ser351Cys	Missense	AD; heterozygous	Likely pathogenic (PM1 + PM2 + PP3 + PP5)	*De novo*	Crouzon syndrome (8946174)	Crouzon syndrome
20	Shortened and bowing bone (−2SD−−3SD)	*COL1A1*	c.1751G > A, p. G584E	Missense	AD; heterozygous	Likely pathogenic (PS2 + PM2 + PP3)	*De novo*	Novel	Osteogenesis imperfecta/Ehlers–Danlos syndrome/Caffey disease
22	Hydrops, AMC	*HBA2*	c.377T > C, p. Leu126Pro	Missense	AD; heterozygous	Likely pathogenic (PM1 + PM2 + PP3 + PP5)	Paternal inherited	HbQS (7070526)	−
24	Shortened and bowing bone (−2SD−−3SD)	*COL1A1*	c.1669−2A > G	Missense	AD; heterozygous	Pathogenic (PS1 + PM2 + PP2 + PP3 + PP4 + PP5)	Maternal inherited	Osteogenesis imperfecta (16705691)	Osteogenesis imperfecta
26	AMC, polyhydramnios	*KLHL40; EMD*	c.1516A > C, p. T506P; c.631C > T, p. R211C	Missense; missense	AR; Homozygous; X-linked; hemizygous	Likely pathogenic (PM1 + PM2 + PP3 + PP5); VUS	Parental inherited; maternal inherited;	Nemaline myopathy type 8 (23746549)	Nemaline myopathy
29	Depressed nasal bridge, turricephaly, brachy-syndactyly of hands and feet	*FGFR2*	c.755C > G, p. Ser252Trp	Missense	AD; heterozygous	Pathogenic (PS1 + PM2 + PP2 + PP3 + PP4 + PP5)	*De novo*	Apert syndrome (7719344)	Apert syndrome
31	Turricephaly, brachy-syndactyly of hands and feet, ARSA, polyhydramnios	*FGFR2; FLNB*	c.755C > G, p. Ser252Trp; c.1076C > A, p. Ala359Glu	Missense; Missense	AD; heterozygous	Pathogenic (PS1 + PM2 + PP2 + PP3 + PP4 + PP5); VUS	*De novo*	Apert syndrome (7719344); Novel	Apert syndrome
35	Dysplasia of thoracic vertebra and ribs, scoliosis	*COL10A1*	c.928delA, p. Arg310fs	Frameshift	AD; heterozygous	Likely pathogenic (PM2 + PM4 + PP3 + PP5)	Maternal inherited	Novel	Schmid metaphyseal chondrodysplasia
36	Micrognathia, polyhydramnios	*BMP2*	c.709C > T, p. Gln237*	Non-sense	AD; heterozygous	Likely pathogenic (PM2 + PM4 + PP3 + PP5)	−	Novel	SSFSC syndrome
41	Depressed nasal bridge, turricephaly, agenesis of corpus callosum, brachy-syndactyly of hands and feet	*FGFR2*	c.755C > G, p. Ser252Trp	Missense	AD; heterozygous	Pathogenic (PS1 + PM2 + PP2 + PP3 + PP4 + PP5)	*De novo*	Apert syndrome (7719344)	Apert syndrome
42	Several cardiac rhabdomyoma, cerebral calcifications and subependymal nodules	*TSC2*	c.1257 + 1G > T, p.?	Splicing mutation	AD; heterozygous	Pathogenic (PS1 + PM2 + PP2 + PP3 + PP4 + PP5)	*De novo*	Tuberous sclerosis (15798777)	Tuberous sclerosis
45	Depressed nasal bridge, turricephaly, ACC, brachy-syndactyly of hands and feet	*FGFR2*	c.755C > G, p. Ser252Trp	Missense	AD; heterozygous	Pathogenic (PS1 + PM2 + PP2 + PP3 + PP4 + PP5)	*De novo*	Apert syndrome (7719344)	Apert syndrome
48	Thoracic hypoplasia, shortened and bowing bone (< −4−−6 SD)	*COL1A2*	c.1360G > T p. Gly454Cys	Missense	AD, heterozygous	Likely pathogenic (PS2 + PM2 + PP3 + PP5)	*De novo*	Osteogenesis imperfecta (17078022)	Osteogenesis imperfecta
50	TOF, tracheoesophageal fistula, unilateral renal agenesis, hemivertebra	*C5orf42*	c.4804C > T, p. Arg1602*	Non-sense	AR, heterozygous	Likely pathogenic (PVS1 + PM2)	−	Joubert syndrome (27158779)	Joubert syndrome
51	Hypospadias	*SRD5A2*	c.680G > A, p. Arg227Gln; c.164T > C, p. leu55Pro	Missense	AR, heterozygous	Pathogenic (PS1 + PM2 + PP2 + PP3 + PP4 + PP5)	Parental inherited	Hypospadias (8768837, 19342739)	Hypospadias
52	Shortened bone (< −6−−8 SD)	*FGFR3*	c.2419T > A, p.*807Argext*101	Terminator codon mutation	AD; heterozygous	Pathogenic (PVS1 + PS2 + PM2)	*De novo*	Thanatophoric dysplasia type I (7647778)	Thanatophoric dysplasia
53	Enlarged polycystic and echogenic kidneys	*ETFDH*	c.1281_1282delAA, p. I428Rfs*6; c.1305T > G, p.Y435*	Frameshift; Non-sense	AR; heterozygous	Likely pathogenic (PVS1 + PM2)	Parental inherited	Novel	
57	Depressed nasal bridge, opened mouth, abnormal posture of hands and feet	*ABCA12*	c.6858delT, p.F2286Lfs*6; c.3456G > A, p.S1152?	Frameshift; splicing mutation	AR, heterozygous	Pathogenic (PVS1 + PS3)	Parental inherited	Ichthyosis (22992804, 23528209)	Ichthyosis
61	Shortened and bowing bone (−2−−3 SD)	*COL1A2*	c.1009G > A, p. G337S	Missense	AD; heterozygous	Likely Pathogenic (PM1 + PM2 + PM5 + PP3)	Maternal inherited	Osteogenesis imperfecta (8829649)	Osteogenesis imperfecta
62	Depressed nasal bridge, turricephaly, unilateral ventriculomegaly, brachy-syndactyly of hands and feet	*FGFR2*	c.755C > G, p. Ser252Trp	Missense	AD; heterozygous	Pathogenic (PS1 + PM2 + PP2 + PP3 + PP4 + PP5)	*De novo*	Apert syndrome (7719344)	Apert syndrome
63	Shortened and bowing bone (−2−−3 SD)	*COL1A1*	c.779G > A, p. G260D	Missense	AD, heterozygous	Likely pathogenic (PM1 + PM2 + PP3 + PP5)	Paternal inherited	Osteogenesis imperfecta (25741868)	Osteogenesis imperfecta
70	Pachygyria, FGR	*ASPM*	c.9032G > A, p.W3011*; c.9862G > T, p.E3288*	Non-sense	AR, heterozygous	Likely pathogenic (PM2 + PM4 + PP3 + PP5)	Parental inherited	Microcephalus (15806441, 19332161, 19770472)	Microcephalus
78	Depressed nasal bridge, turricephaly, dysplasia of septi pellucidi and cerebellar vermis, brachy-syndactyly of hands and feet	*FGFR2*	c.755C > G, p. Ser252Trp	Missense	AD; heterozygous	Pathogenic (PS1 + PM2 + PP2 + PP3 + PP4 + PP5)	*De novo*	Apert syndrome (7719344)	Apert syndrome
79	Shortened bone (−2−−3 SD), polyhydramnios	*FGFR3*	c.1138G > A, p. Gly380Arg	Missense	AD; heterozygous	Pathogenic (PS2 + PS3 + PM2 + PP1 + PP3)	*De novo*	Achondroplasia (8078586)	Achondroplasia
83	Bilateral anophthalmia	*SOX2*	c.310G > T, p. Glu104*	Non-sense	AD; heterozygous	Pathogenic (PS2 + PM2 + PP2 + PP3 + PP4 + PP5)	*De novo*	Microphthalmia (18385794)	Microphthalmia
90	Unilateral polycystic kidney and ureterocele in the bladder	*SALL1*	c.598C > T, p. L200F	Missense	AD, Heterozygous	Likely pathogenic (PS2 + PM2 + PP3)	*De novo*	Townes–Brocks syndrome type I (20520617)	Townes–Brocks syndrome
91	AMC	*ZC4H2*	c.162delT, p. Leu55fs	Frameshift	X-linked inherited, heterozygous	Pathogenic (PVS1 + PS2 + PM2 + PP2+ PP3 + PP4 + PP5)	*De novo*	Novel	Wieacker–Wolff syndrome
92	Persistent hyperplastic primary vitreous	*NDP*	c.240_243del, p. Phe81fs	Frameshift	X-linked inherited, hemizygous	Pathogenic (PVS1 + PM2 + PM4 + PP3 + PP5)	*De novo*	Novel	Norrie disease
94	Hypospadias	AR*	c.170_172dupTGC, p. Leu57dup	Microduplication mutation	X-linked inherited	Likely pathogenic (PS3 + PM6 + PP5)	*De novo*	Hypospadias (25500996)	Hypospadias

ACMG, American College of Medical Genetics and Genomics; AMC, arthrogryposis multiplex congenital; ARSA, aberrant right subclavian artery; ACC, agenesis of corpus callosum; AD, autosomal dominant; AR, autosomal recessive; FGR, fetal growth restriction; IAA, interrupted aortic arch; TOF, Tetralogy of Fallot; WES, whole-exome sequencing; VSD, ventricular septal defect. *AR is the name of the gene.

In 94 cases, the diagnostic yield of cases with anomalies affecting one anatomic system was 40% (24/60), and that was 38.2% (13/34) in cases with anomalies involved in two or more anatomic systems. The diagnostic yield of cases with fetal abnormalities affecting one anatomic system was higher than that involved in two or more anatomic systems, while there was no significant difference (*p* = 0.87). Of the 60 cases affected with one anatomic system, 19 cases had skeletal abnormalities, with the highest diagnostic yield of 68.4% (13/19). Cardiac abnormalities were detected in 12 cases. Isolated cardiac abnormalities, such as transposition of the great arteries (case 8), double outlet left ventricle, pulmonary valve dysplasia (case 23), and common arterial trunk (case 82), did not reveal pathogenic variants. Only one case (case 3) with a ventricular septal defect and interrupted aortic arch (type B) detected the pathogenic variant *ANKRD11*. There were 11 cases with genitourinary abnormalities. Two cases with polycystic and echogenic kidneys had variants in *ETFDH* (case 53) and *SALL1* (case 90), while another two cases with similar phenotypes had a biparentally inherited missense variant in *PKD1* of uncertain significance. Two of three hypospadias cases had pathogenic variants in *SRD5A2* (case 51) and *AR* (case 94). No pathogenic variant was detected in the severe case with common cloacal deformity. Six cases involved the central nervous system. Only one fetus with pachygyria had a non-sense variant in *ASPM* (case 70). The remaining cases, including bilateral ventriculomegaly and Dandy–Walker malformation (case 11), lissencephaly (case 39), and pachygyria (case 81), did not show detectable pathogenic variants. Of the six cases with head anomalies, three variants were found: two had abnormalities in the eyes with bilateral anophthalmia in *SOX2* (case 83) and persistent hyperplastic primary vitreous in *NDP* (case 92), and one had micrognathia in *BMP2* (case 36). Another three cases with microphthalmia or microtia did not show pathogenic variants. Four cases with arthrogryposis were screened and categorized as neuromuscular abnormalities, and the diagnostic yield was 50% (2/4) with variants in *KLHL40* (case 26) and *ZC4H2* (case 91). One hydrops fetalis and one case with an anomaly of the abdominal wall did not show a pathogenic variant.

## Discussion

In our study, the diagnostic yield of WES in anomalous fetuses with normal karyotype and CMA results was 39.4%. A genetic diagnosis was nearly nine times more frequent in trios than in proband-only cases. In fetuses with abnormalities in two or more anatomic systems, the diagnostic yield of cases was 38.2% compared with 40% if a single anatomic system was involved. Among the cases involving one anatomic system, the skeletal and neuromuscular systems and head anomalies had higher diagnostic yields.

The strength of our study was that it included various kinds of fetal abnormalities and added or compared the postnatal phenotypes with prenatal phenotypes for the genetic interpretation. The diagnostic yield in our study was higher than that in other studies. Lord et al. (2019) found that 8.5% of pathogenic variants were detected by WES in unselected fetuses with structural abnormalities, including increased nuchal translucency (NT). Fu et al. (2018) and Petrovski et al. (2019), respectively, showed 24% and 10.3% diagnostic yields by WES in fetuses with structural abnormalities with normal karyotype or CMA results. In highly selected cases, such as terminated or miscarried fetuses (Sa et al., 2018) or fetuses with anomalies in the specific anatomic system (Han et al., 2020), the diagnostic rate of pathogenic variants was up to 53.3–88.9%. We hypothesized that the highly selected cases of skeletal dysplasia and fetuses with severe abnormalities led to the higher diagnostic yield of WES, as was shown in our prospective cohort.

Proband-only and trio sequencing were included in our research. Parents decided whether to provide the blood sample after genetic counseling according to their willingness. Both proband-only and trio groups included fetal anomalies in one and two or more anatomic systems, and a higher proportion of cases with abnormalities affecting multisystems was found in the proband-only group. There were 15 cases (15/41, 46.3%) with abnormalities in two or more anatomic systems among the proband-only group, while 19 cases (19/53, 35.8%) were in the trio group; however, the trios had a higher diagnostic yield. The proportion of skeletal abnormalities in the trio group led to a higher rate of genetic variants than that in the proband-only group.

The genetic diagnosis of congenital heart defects and central nervous system defects was challenging because of genetic and phenotypic heterogeneity, while the condition was different in the skeletal system. The fetuses with skeletal anomalies had the highest detection yield in WES. Skeletal dysplasia is a heterogeneous group of disorders consisting of 436 entities and 364 known genes (Bonafe et al., 2015). Osteogenesis imperfecta with short and angulated bones, thanatophoric dysplasia with extremely short bones, and achondroplasia with short bones are the most common prenatal diagnoses (Vora et al., 2020). Despite these disorders, Apert syndrome with turricephaly and brachy-syndactyly of hands and feet was also commonly observed in our cohort. These kinds of diseases are associated with the *COL1A1* or *COL1A2*, *FGFR3*, and *FGFR2* variants; however, cases of short bones do not always find exact diagnostic genetic variants. Some cases with short bones (−2 to −3 SD) had variants of uncertain significance (VUS), such as *COL11A2* of uncertain significance in case 6. The mother terminated two pregnancies with skeletal anomalies and eventually found a VUS that was useless for the guidance of the next pregnancy.

Except for the skeletal system, the neuromuscular system and head anomalies had higher diagnostic yields. In the neuromuscular system, eight fetuses with arthrogryposis multiplex congenita (AMC) of different genotypes were found. Four diagnostic genetic variants were detected in *HBA2*, *KLHL40*, *MYH3*, and *ZC4H2*. Two had VUS in *FBN3* and *TTN*. As previously reported (Hall et al., 2019), over 400 known conditions are associated with AMC. The etiology of arthrogryposis is complicated and may be related to the decrease in fetal movement caused by the abnormal central nervous system, muscle and connective tissues, lack of exercise space, maternal disease, environmental factors, and viral infection (Haliloglu and Topaloglu, 2013). Only 30% of AMC cases can find clear pathogenic genes (Skaria et al., 2019). In our study, 50% of genetic variants were found, which suggested that WES would be helpful for prenatal AMC cases. In addition, of the six cases with one anatomic system affected by head anomalies, two cases with abnormalities in the eyes and one with mandibular anomaly detected genetic variants. The major single-gene *SOX2* variant was found in a fetus with bilateral anophthalmia, which accounted for 10–15% of all anophthalmia and microphthalmia cases (Ragge et al., 2007). Although *SOX2* in anophthalmia, persistent hyperplastic primary vitreous in *NDP*, and micrognathia in *BMP2* were detected in this study, up to 60% of cases with underlying genetic causes remained undetermined.

The consequences of genetic variant might relate to pregnancy management, including both prognoses of the affected pregnancy and recurrence risk of continuing pregnancy. According to our study, a diagnostic genetic variant might be more likely to be identified in selected cases, especially skeletal anomalies as that was reported in the previous study and neuromuscular or head anomalies as shown in ours. It would be helpful for variant interpretation and might improve the diagnostic yield if WES was performed for both parents and fetuses. The multidisciplinary meeting was held among molecular geneticists, maternal–fetal medicine specialists, and an expert in obstetric ultrasound before clinical counseling. The results related to clinical phenotypes detected by ultrasound would be reported back, not secondary findings. In general, the pediatric geneticist was also included in the multidisciplinary conference, while it was not in this study, which was one of our limitations. Although 88 of 94 pregnancies were terminated, and the potential prognosis of these affected pregnancies was unknown, the genetic variants would effectively predict the recurrence risk of continuing pregnancy according to the types of inherited variants.

There are other two limitations in our study. First, our hospital is a tertiary level referral center, and abnormal cases diagnosed in downtowns are transferred to our hospital, which leads to a relatively high detection rate in rare abnormal cases, such as osteogenesis imperfecta and Apert syndromes. Second, although a large number of fetuses have structural anomalies, only a small number of parents chose to participate in our research, and cases eligible for our study included proband-only and trio groups depending on parents’ willingness to provide blood samples, which might lead to inevitable selection bias. The proportion of skeletal abnormalities in the trio group might have led to a higher diagnostic yield than that in the proband-only group.

In conclusion, prenatal WES can improve the genetic diagnostic yield of anomalous fetuses with accurate phenotypes with normal karyotype and CMA results and expand the information for the prenatal genotype–phenotype spectrum. With the utility of WES, parents will gain information on the guidance and assessment of recurrence risk for the subsequent pregnancy. A preimplantation genetic diagnosis can be performed for cases with specific inherited variants.

## Data Availability Statement

The datasets presented in this study can be found in online repositories. The names of the repository/repositories and accession number(s) can be found below: NCBI BioProject, accession no: PRJNA713979.

## Ethics Statement

The studies involving human participants were reviewed and approved by The First Affiliated Hospital of Sun Yat-sen University. The patients/participants provided their written informed consent to participate in this study. Written informed consent was obtained from the individual(s) for the publication of any potentially identifiable images or data included in this article.

## Author Contributions

MH and LD mainly conducted the design of this study and analysis of the results. MH mainly wrote and submitted the manuscript. HX provided the prenatal cases. LZ and YG helped with the pathogenic examination and sample collection. TL and JZ provided a few cases. DC was responsible for the sample delivery. All authors contributed to the article and approved the submitted version.

## Conflict of Interest

DC was employed by the company Guangzhou Kingmed Diagnostics Group. The remaining authors declare that the research was conducted in the absence of any commercial or financial relationships that could be construed as a potential conflict of interest.

## Footnotes

1 http://www.omim.org

2 https://www.genecards.org

3 http://www.ncbi.nlm.nih.gov/clinvar

4 http://www.hgmd.org
